# Acknowledgement

**Published:** 1893-06

**Authors:** 


### Acknowledgement.

It is due to the publishers of the Ohio Journal of Dental Science to acknowledge our indebtedness to them for their admirable cut of the late Dr. George Watt, which appears as the frontispiece of the May Register.

